# Novel virus, atypical risk group: understanding young adults in college as an under-protected population during H1N1 2009

**DOI:** 10.1371/currents.flu.ce9ad5d14c88ccf5877b9cf289a41eaf

**Published:** 2012-12-20

**Authors:** Karl Maier, Jennifer Berkman, David Chatkoff

**Affiliations:** Salisbury University; DirectorSalisbury University; University of Michigan - Dearborn

## Abstract

Objective: To examine illness/vaccination perceptions of and intentions to vaccinate for seasonal influenza (SI) and 2009 H1N1 in the college setting. Participants: 1190 adults [M=23.5 years (SD=9.5)] from a university in the North-Eastern U.S. Methods: We deployed a web-based survey via campus email just prior to the 2009 H1N1 vaccine release. Results: Younger adults (18-24 years) had lesser understanding of the difference between influenza types, and they reported less regular and current SI vaccination compared to older adults (25-64 years). Younger respondents perceived lesser likelihood of illness from, but attributed greater severity to H1N1 versus SI. Regularity of SI vaccination and perceived vaccine efficacy were the strongest predictors of intent to vaccinate against H1N1, followed by perceived likelihood of illness and confidence in what experts know about vaccine safety. Conclusions: Young adults in college may require additional information during novel influenza pandemics. Measuring perceptions and past vaccination behaviors may facilitate targeting of preventive efforts in the college setting.

## Introduction

A defining characteristic of the 2009 and prior 1 H1N1 influenza pandemics was the disproportionate impact on young adults who are typically less susceptible to complications from seasonal influenza (SI). The young adult college population therefore served as a valuable model for understanding the H1N1 virus and illness characteristics early in the 2009 pandemic 2. Prior to this, college samples have been used for testing SI preventive behaviors 3 and to investigate relations of risk perception to subsequent vaccination for SI 4.

Despite this and national media attention to the risk for young adults, there was a strikingly low degree of protection across the college population in the United States (U.S.) during H1N1 2009. During the pandemic, a national survey of colleges by the American College Health Association (ACHA) indicated that the average H1N1 vaccine uptake rate was approximately 8%. Furthermore, only 61% of participating institutions had acquired vaccine^5^. This is in contrast to coverage estimates of all U.S. adults ranging from about 9% to 34% by state early in the pandemic^6^. Given the clear risks for the college population in 2009, the relatively low coverage for this group underscores the need to better understand the determinants of vaccination in order to enable campus healthcare and administrators to respond optimally to future novel threats. Institutions of higher education have been engaged in pandemic influenza planning for several years^7^, and the process is one that evolves over time as new information becomes available. In addition to more fully appreciating the impact of H1N1 on the college population in 2009, understanding perceptions during that novel influenza pandemic may also prove useful for anticipating and managing future novel threats in this population as novel variants of influenza continually emerge^8^.

Other than focal distribution problems that may partially explain the observed coverage rates, we are not aware of any research to date on perceptual determinants of 2009-2010 vaccination behavior in college populations. There is, however, a sizeable literature now from the general U.S. population suggesting an unfavorable climate for accomplishing high rates of coverage nationally in 2009. Although about half of the population intended to receive the novel vaccine early in the pandemic, many adults had diminishing concerns about getting sick with H1N1, and had concerns over the safety and potential side effects of the vaccine^9^. Other surveys revealed a sentiment that the H1N1 vaccine was not as safe or worth the expense as the SI vaccine 10 11. In contrast, past SI vaccinationwas associated with greater intention to utilize the H1N1 vaccine just prior to the pandemic in 2009^12^. With regard to SI prior to 2009, perceptions of low risk and low perceived vaccine effectiveness, as well as concern of getting ill from the vaccine, have been associated with low SI vaccine uptake among younger and mid-life adults^4^ ^13^.

It is unknown if the available findings generalize either to pandemic H1N1, or to the population of young adults in college. Although a range of perceptual, attitudinal, and behavioral variables may explain the lower than expected coverage across college campuses, factors other than past vaccination^12^ have not been analyzed in predictive/explanatory models of pandemic H1N1 vaccination in any population. We therefore measured perceptions related to the seasonal and H1N1 influenzas as well as past vaccination behavior in a college sample, and examined the association of these factors with self-reported likelihood of vaccinating for H1N1 just prior to the 2009 vaccine release. Although we did not assess actual H1N1 vaccination behavior among survey respondents, behavioral intentions are associated with vaccination behavior^14^ and the initiation of health behaviors in general^15^.

## Methods

In the two weeks immediately prior to H1N1 vaccine distribution to the region of this study sample (October, 20 to November 2, 2009), a survey web link was sent via email to the entire population of approximately 8000 students and 700 employees affiliated with Salisbury University, a mid-sized university in Salisbury, Maryland, located in the North-Eastern U.S. The web link alternately deployed a 10-minute survey about either SI or H1N1 influenza, with informed consent obtained on the first page. A chance to win one of ten, $20.00 gift cards to the campus bookstore was offered for completing the survey. Of 1300 surveys accessed, 1190 were fully or largely completed. The protocol was approved by the Salisbury University Committee on Human Research.

Respondents were ages 18-64 years (M=23.5, SD=9.5; 77% female; 87% Caucasian). Consistent with H1N1 2009 risk stratification by the Centers for Disease Control and Prevention (CDC), both younger (18-24yrs) and older (25-64yrs) adults were examined. The young adult group (n=879; 74% of the sample) consisted of 94% undergraduates, and 79% females. Among the older adult group (n=302; 26% of the sample), 78% were staff, faculty, or administrators, and 73% were female. Most respondents (95%) reported having a permanent address in Health and Human Services region 3 over the past 12 months. Young adults reported their healthcare utilization during the semester as: “On Campus” (40%); “Off Campus (local)” (27%); “Off Campus (not local)” (23%); “I usually do not seek health care if I need it” (10%).

**Measures. **H1N1 vaccine coverage was determined for comparability with other populations (doses administered to the general campus community divided by the campus population).

***Survey. ***Survey respondents indicated which type of influenza they believed had the most severe symptoms (Regular seasonal flu; H1N1 ("swine") flu; No difference in symptoms; I dont know). Participants also endorsed reasons for not getting vaccinated (see Table 4 for items used). Several items are listed below that measured self-reported perceptions and behavior related to the SI or H1N1 illness and vaccine on a rating scale of 0 to 10, with 0 indicating lesser understanding, likelihood, severity, or confidence.

***Illness Perception Items:*** Understand difference: Please rate your understanding of the difference between the regular seasonal flu and the new H1N1 ("swine") flu.

Likelihood of illness: How likely do you think you are to get sick with the _____ flu this year?

Illness severity: How severe do you think the illness would be for you if you got sick with the _____ flu?

***Vaccine Perceptions, Behavior and Intention Items. ***Likelihood of vaccination: If you did not already get a vaccine for this year's _____ flu, how likely are you to get one for the _____ flu if one is available?

Vaccine efficacy: If you were to get a vaccine for the _____ flu this year, how much do you think it would actually prevent you from getting sick with the _____ flu?

Effectiveness confidence: Rate your confidence in what health experts understand about the effectiveness of the _____ flu vaccine.

Safety confidence: Rate your confidence in what health experts understand about the safety of the _____ flu vaccine.

Vaccination history: IN PAST YEARS, how regularly have you received a flu shot (vaccine) for the seasonal flu? *(Never had one in my life; Rarely; Occasionally - some years but not others; Usually - most years but not always; Every year)*

***Statistical Analyses. ***Group differences on perceptions and frequency data were examined using t-tests for independent samples, and Chi Square analyses, respectively. Pearson correlations were used to examine associations between likelihood of getting vaccinated and influenza-related perceptions and behaviors. Independent associations of each continuous variable with intent to vaccinate (continuous variable) were tested using ordinary least squares (OLS) hierarchical regression. This allowed for determination of each variable’s unique relation with intent to vaccinate above and beyond the effect of other factors examined.

## Results

In this sample, 296 respondents (28%) reported having obtained the current SI vaccine by the time of the survey, with a greater proportion of those in the 25-64 year age group (41%) adopting the vaccine relative to the younger group (23%) (1% of the younger group reported “not sure” to this question) [χ^2^(2, n=1065)=36.52, p<.001]. Current SI vaccination was associated with a greater self-reported likelihood of seeking H1N1 vaccination (M=6.55, SD=3.49) compared to no current SI vaccination (M=3.54, SD=3.46), F(1, 506)=78.68 (p<.001), regardless of age. Respondents described the regularity of obtaining the SI vaccine in the past as: 16% “every year”; 12% “usually (most years but not always)”; 14% “occasionally (some years but not others)”; 23% “rarely”; and 35% “never had one in my life”. Those in the older group reported more regular annual vaccination [χ^2^(4, n=1065)=51.91, p<.001].

There was no significant difference between the proportion of respondents who indicated that they “definitely will” get a vaccine for H1N1 (17%; n=85) compared to SI (16%; n=61) [χ^2^(1, n=897)=.060, p=.807]. The actual rate of H1N1 vaccine coverage throughout the flu season from on-campus distributions in the immediate population sampled was 9%, which was similar to the average of colleges nationally^5^.

**Descriptive Findings of Illness and Vaccine Perceptions. **Across all respondents there was moderate understanding of the difference between the two influenza types (M= 6.4, SD=2.6), with 15% reporting that they “understand very well”. However, younger adults reported less understanding of the difference (M=5.98, SD=2.51) than older adults (M=7.38, SD=2.36), t(1065)=-8.15, p<.001. Forty nine percent of respondents perceived H1N1 to have the “most severe symptoms” compared to 11% for SI. “No difference in symptoms” was reported by 31%, and 9% reported that they “don’t know” which is more severe [χ2(3, n=1073)=454.54, p<.001]. Perceptions did not differ by age [χ2(3, n=1064)=4.56, p=.21].

Younger respondents viewed themselves as less likely to become ill with H1N1 compared to SI, but perceived that the H1N1 illness would be more severe for them than SI (see Table 1). However, there was no mean difference in likelihood of young adults to vaccinate for SI versus H1N1. Young adults also reported less confidence in the safety and effectiveness of the H1N1 vaccine compared to the SI vaccine (see Table 1). Compared to older adults, younger adults did not report any greater intention to vaccinate for H1N1, despite rating H1N1 with a higher level of severity than SI (see Table 2).

**Table 1. Illness and Vaccine Perceptions by Influenza Type and their Independent Contributions in Predicting Intention to Vaccinate among Young Adults, Age 18 to 24.** Note. Results are based on 1190 responses from a survey of students and faculty/staff at a university in the North-Eastern U.S., October 20– November 2, 2009 just prior to H1N1 vaccine availability. Mean group differences were examined using t-tests for independent samples. Independent associations of each variable with intent to vaccinate were tested using hierarchical linear regression. All variables (except "Understand difference," which is categorical) are ratings on a scale of 0 to 10. Seasonal influenza models exclude those currently vaccinated for seasonal influenza. *R* ^2^= proportion of variance accounted for; *β* = standardized regression coefficient. Full Model = regression model with all predictors included in the model. Statistical significance of group difference or regression parameter indicated = *p≤.05; **p≤.01; ***p≤.001. ^a^ *F*(7,266)=42.37; ^b^ *F*(7,346)=39.66.

							Intention to Obtain Vaccine for:				
	Seasonal Influenza			H1N1 Influenza			Seasonal Influenza			H1N1 Influenza	
	N	M(SD)		N	M(SD)		*R* ^2^	*β*		*R* ^2^	*β*
**Illness Variables**							.19***			.13***	
Understand difference		*NA*			*NA*		.00	-.05		.00	.00
Likelihood of illness	374	3.95(2.23)		371	3.43(2.16)***		.01*	.11*		.03***	.18***
Illness severity	375	4.73(2.18)		373	5.24(2.11)***		.02**	.16**		.01	.08
											
**Vaccination Variables**							.34***			.32***	
Likelihood of vaccination	275	4.04(3.67)		285	3.68(3.46)		*NA*			*NA*	
Vaccination history		NA		NA			.11***	.37***		.09***	.29***
Vaccine efficacy	375	5.98(2.41)		370	5.50(2.44)**		.06***	.31***		.11***	.41***
Confidence effective	374	6.53(1.97)		370	5.26(2.30)***		.00	.06		.00	-.06
Confidence safety	375	6.73(1.95)		372	5.18(2.40)***		.00	.04		.01*	.15*
Full Model							.53***^a^			.45***^b^	

**Table 2. H1N1 Illness and Vaccine Perceptions by Age:** Note. All variables are ratings on a scale of 0 to 10. No sex differences were found for either age group or with all ages combined.

	Ages 18-24			Ages 25-64				
**Illness**	N	M(SD)		N	M(SD)		Diff(95% CI	p≤
Likelihood of illness	371	3.43(2.16)		147	3.20(1.83)		.23(-.16-.63)	.252
Illness severity	373	5.2(2.11)		146	4.46(1.86)		.78(.39-1.17)	.001
**Vaccination**								
Likelihood of vaccination	366	4.26(3.64)		145	4.63(3.88)		-.37(-1.09-.34)	.307
Vaccine efficacy	370	5.50(2.44)		147	5.95(2.28)		-.45(-.91-.01)	.053
Confidence effective	370	5.26(2.30)		149	5.30(2.75)		-.05(-.51-.42)	.848
Confidence safety	372	5.18(2.40)		150	4.99(2.83)		.20(-.29-.68)	.424

**Explanatory Findings of Illness and Vaccine Perceptions**

Zero-order correlations among perceptions, past behavior, and likelihood of obtaining vaccine for the full sample are presented in Table 3. The unique associations of illness and vaccine variables with likelihood of vaccination are reported in Table 1 for young adults. After controlling for the effects of other variables, a greater regularity of SI vaccination was strongly associated with a greater intent to be vaccinated for each influenza type. The perceived ability of the vaccine to prevent illness was likewise strongly associated with vaccination likelihood for both types of influenza, but the association was strongest for H1N1. Perceived likelihood of illness was modestly associated with a greater likelihood of obtaining the vaccine for each type of influenza. Finally, confidence in what health experts understand about the safety of the vaccine explained a small amount of variance in likelihood to obtain the H1N1 but not the SI vaccine.

**Table 3. Correlates of Illness and Vaccine Perceptions with Intention to Vaccinate, by Influenza Type, Full Sample:** Note. Correlation coefficients in the lower left quadrant are based on survey responses pertaining to seasonal influenza; Correlation coefficients in the upper right quadrant are based on survey responses pertaining to H1N1 influenza. Correlations for seasonal influenza exclude respondents currently vaccinated for seasonal influenza. *p<.05; **p<.01; ***p<.001.

	Intent	Under- stand difference	Likeli- hood of illness	Illness severity	Vacci- nation history	Vaccine efficacy	Confi- dence effective	Confi- dence safety	Intent
			***H1N1 Influenza***						
**Understand difference**	.06		.01	-.05	.18***	.19***	.18***	.12**	.09*
**Likelihood of illness**	.32***	.06		.34***	.09*	.09*	.11*	.12**	.29***
**Illness severity**	.37***	-.03	.47***		.14**	.22***	.17***	.15**	.30***
**Vaccination history**	.69***	.11*	.20***	.19***		.28***	.20***	.14**	.46***
**Vaccine efficacy**	.58***	.10	.21***	.25***	.37***		.58***	.51***	.57***
**Confidence effective**	.41***	.14**	.12*	.18**	.25***	.56***		.80***	.41***
**Confidence safety**	.39***	.09	.13*	.18**	.25***	.52***	.79***		.41***
***Seasonal Influenza***									

**Reasons for not Vaccinating.**Fewer respondents from the H1N1 survey (24%; n=121) indicated that they will definitely not get vaccinated for H1N1, relative to those completing the SI survey (30%; n=114) [χ2(1, n=897)=4.40, p=.04]. Table 4 indicates a wide distribution of reasons (mode=1 reason) endorsed for not getting vaccinated for each influenza type, with differences observed for availability, concern about the illness, and exposure. The leading reason for not obtaining a vaccine for each influenza type was a fear of side effects.

**Table 4. Reasons for not Getting Vaccinated, Full Sample:** Note. Question stem: If you plan NOT to get vaccinated for the ______ flu, please indicate why (question worded with either “seasonal” or “H1N1”, depending on survey version. % = percent who endorsed that reason among respondents answering the question. p = statistical significance for Chi Square test of frequency differences between influenza types.

Reason Endorsed	H1N1 Influenza			Seasonal Influenza		
	% of 321	(n)		% of 266	(n)	p≤
Fear of side effects from the _____ shot/vaccine	34%	(109)		32%	(84)	0.082
**Availability of the _____ shot/vaccine**	**29%**	**(92)**		**19%**	**(51)**	**0.001**
I don't think I will get the _____	22%	(71)		26%	(68)	0.923
Fear of getting the flu from the _____ shot/vaccine	19%	(61)		22%	(59)	0.974
**I'm not concerned about the _____ **	**19%**	**(60)**		**33%**	**(87)**	**0.011**
Cost of the _____ shot/vaccine	19%	(60)		22%	(59)	0.950
I don't think I am in a high risk category for getting the _____	15%	(49)		22%	(58)	0.290
I don't think the _____ shot/vaccine will actually work	15%	(47)		22%	(58)	0.205
Fear of shots in general	13%	(41)		17%	(46)	0.491
**I think I will be exposed enough to the ___ virus in everyday life to build immunity **	**6%**	**(20)**		**12%**	**(33)**	**0.054**
**I don't care if I get the _____ **	**4%**	**(14)**		**15%**	**(41)**	**0.001**
Philosophical or religious reasons	2%	(6)		3%	(7)	0.742
I think the _(other) vaccine I got (or plan to get) might protect me from the _____	2%	(5)		2%	(4)	0.770
I may intentionally expose myself to those sick with the _____ to build immunity	1%	(3)		2%	(4)	0.678

## Discussion

Despite the increased risk for young adults, vaccine coverage rates for this sample and colleges nationally^5^ suggest that young adults in college were vaccinated less than the general public during the 2009 H1N1 pandemic. Given this differential, even in a climate of unusually high risk for young adults, the present findings suggest some behavioral and perceptual factors for college healthcare professionals to consider in addressing future pandemic threats.

Although there were no age differences in self-reported likelihood of vaccination for H1N1 in this sample, younger adults were not as likely as older adults from this campus community to have been vaccinated for SI, and were less likely to report a history of regular vaccination. A similar finding for SI vaccination was found previously across other college samples^4^. This is important given the results of the present study and of a national survey of the general adult population^12^ that vaccination history was among the strongest predictors of self-reported likelihood of obtaining the H1N1 vaccine.

Adults aged 18-49 in the general population have also been found to vaccinate less than those aged 50-64, who in turn vaccinate less than those 65 and older^12^, even when high risk medical conditions are present^16^. These findings, along with our data, suggest a continuum of vaccination utilization among adults that increases with age. As well, a significant number of students in this sample reported lesser influenza understanding and not using health care when needed, despite it being freely available. This is a limited sample, but could suggest that some young adults in college may be particularly difficult to reach and protect during an influenza pandemic^17^. Conversely, the fact that over half of the sample in this study had rarely or never obtained an SI vaccine suggests that there is considerable potential for prevention efforts aimed at increasing regular vaccination.

Success at increasing regular SI vaccination among young adults could potentially improve the likelihood of pandemic vaccine acceptance in this population, given the available cross-sectional data reported here and by others^12^. Although these correlational findings do not in themselves indicate causation, our data do establish that regular vaccination is associated with more favorable views of both seasonal and pandemic vaccines. It is reasonable to suggest then that accomplishing an increase in regular seasonal vaccination in the population would be associated with improved attitudes toward and utilization of a future novel vaccine.

The available prospective studies of risk perception variables do suggest a causal role of perceptual factors in vaccination behavior that are consistent with the present findings and health behavior models^4^ ^18^. In this study, perceived risk of illness was associated with greater likelihood of H1N1 vaccination, yet perceived illness severity, another indicator of risk^18^, was not . Although this could suggest that perceived severity is not an important determinant of novel pandemic vaccination, the responses on our measure indicate modest perceptions of severity in this sample (see Tables 1&2). It is notable that perceived severity was predictive of vaccination intent with a putatively more severe virus in a survey about a hypothetical H5N1 pandemic in a general population sample from The Netherlands^19^. This, and the differential findings for perceived severity predicting likelihood of adopting a novel vaccine in the present study, underscore the importance of measuring multiple facets of risk that may yield disparate conclusions^20^.

In addition to determining who is likely to get vaccinated, the various perceptual factors examined here may conversely be useful for determining the proportion of the population that does not intend to vaccinate, particularly when several factors are considered simultaneously^19^. This could potentially facilitate the targeting of non-pharmacological preventive efforts on campus to those not willing to receive a pandemic vaccine. Likewise, the low rate of SI vaccination among young adults may further highlight the need for campus health initiatives to support preventive efforts other than vaccination that have shown promise in college populations for managing influenza, such as protective mask use and hand hygiene^3^.

Another consideration in reaching the college population pertains to the reasons individuals may have for not obtaining a vaccine. A variety of reasons were provided by respondents, however, the reason most often endorsed in this study was fear of side effects. This is consistent with the available findings for SI in other adult samples^11^ ^21^. Also, given the public safety concerns related to the pandemic vaccine^9^ ^10^, it is not surprising that greater confidence in what health experts understand about the safety of the vaccines was associated with greater intent to receive vaccine for H1N1 but not SI in this study. This aligns with findings from a national sample of (presumably) older adults that H1N1 vaccination was highest among those identifying employers and healthcare providers as their most influential sources of information^10^. Given the influence that healthcare providers may have on vaccination behavior^10^ ^14^, it may be important for clinicians to collaboratively address with their student patients a range of individual safety concerns and informational needs within the context of available scientific information on risk and safety^22^.

On a broader public health level, the importance of susceptibility, vaccine safety, and vaccine efficacy as predictors of vaccination intent, points to the value of having adequately tested vaccines available. In addition to perceptions of a novel vaccine, the actual accessibility of it is also important. In one survey of the general population and SI vaccination, respondents who intended to vaccinate but failed to follow through reported simply “not getting around to being vaccinated” as their primary reason. Therefore, despite the inherent challenges of making pandemic vaccines widely available, the extent to which available doses can be easily accessed may play an important role in maximizing uptake among those intending to vaccinate. In addition, the lesser understanding of the differences between SI and H1N1 observed in the present study suggests that educational efforts providing even basic accurate information using appropriate levels of detail^22^ might be beneficial. On a college campus this may rather easily extend beyond individual patient contact within the student health center to the broader campus community by way of campus media and communications.

## Limitations and Conclusions

This study is based on a limited sample derived from one region; however, similarity between coverage rates reported here and nationally suggests that the present findings may generalize to the broader young adult college population of the U.S. The present findings indicate a need to better educate and protect students during high-risk influenza pandemics. In this regard, young adults in college were generally less understanding of differences in the types of influenza than older campus members, but were nevertheless concerned about their risk and protection from H1N1. Perception of vaccine efficacy was one of the strongest predictors of vaccination intent for H1N1 among young adults, whereas confidence in what health experts know about the effectiveness of the vaccine was not related. Young adults therefore may give considerable weight to their own sense of how much they would benefit from adopting a novel vaccine, and less to authoritative information about effectiveness.

We also note that some effect sizes in the explanatory models (Table 1), reflecting independent associations of perceptions with likelihood of vaccination, ranged from small to moderate. These factors therefore differ in relative importance for determining intent to vaccinate, but the clinical significance of these effects on real outcomes is unclear given that actual behaviors were not assessed. The clinical significance of the observed effects is also difficult to gauge because of the overall lack of applied research in this area. We emphasize, however, that there were large effects observed on intent to vaccinate when all factors were considered together. This might suggest that educational initiatives for influenza prevention may be most effective if multiple attitudes about novel illness and vaccine are targeted simultaneously. This also speaks to the potential utility of developing predictive models whereby perceptual and behavioral factors are evaluated simultaneously to identify those who are likely and not likely to vaccinate^19^ in order to better target vaccination and non-pharmaceutical preventive efforts. Although data from multiple samples would be needed to develop such a model, the present findings alone provide a starting point for better informing campus-wide and clinic-based initiatives designed to prepare the college population for future novel influenza threats.
